# A machine learning model for predicting patients with major depressive disorder: A study based on transcriptomic data

**DOI:** 10.3389/fnins.2022.949609

**Published:** 2022-08-08

**Authors:** Sitong Liu, Tong Lu, Qian Zhao, Bingbing Fu, Han Wang, Ginhong Li, Fan Yang, Juan Huang, Nan Lyu

**Affiliations:** ^1^The National Clinical Research Center for Mental Disorders & Beijing Key Laboratory of Mental Disorders, Beijing Anding Hospital, Capital Medical University, Beijing, China; ^2^Advanced Innovation Center for Human Brain Protection, Capital Medical University, Beijing, China; ^3^Department of Thoracic Surgery, The Second Affiliated Hospital of Harbin Medical University, Harbin, China

**Keywords:** major depressive disorder, machine learning, random forest, artificial neural network, bioinformatics analysis

## Abstract

**Background:**

Identifying new biomarkers of major depressive disorder (MDD) would be of great significance for its early diagnosis and treatment. Herein, we constructed a diagnostic model of MDD using machine learning methods.

**Methods:**

The GSE98793 and GSE19738 datasets were obtained from the Gene Expression Omnibus database, and the limma R package was used to analyze differentially expressed genes (DEGs) in MDD patients. Gene ontology (GO) and Kyoto Encyclopedia of Genes and Genomes (KEGG) enrichment analyses were performed to identify potential molecular functions and pathways. A protein-protein interaction network (PPI) was constructed, and hub genes were predicted. Random forest (RF) and artificial neural network (ANN) machine-learning algorithms were used to select variables and construct a robust diagnostic model.

**Results:**

A total of 721 DEGs were identified in peripheral blood samples of patients with MDD. GO and KEGG analyses revealed that the DEGs were mainly enriched in cytokines, defense responses to viruses, responses to biotic stimuli, immune effector processes, responses to external biotic stimuli, and immune systems. A PPI network was constructed, and CytoHubba plugins were used to screen hub genes. Furthermore, a robust diagnostic model was established using a RF and ANN algorithm with an area under the curve of 0.757 for the training model and 0.685 for the test cohort.

**Conclusion:**

We analyzed potential driver genes in patients with MDD and built a potential diagnostic model as an adjunct tool to assist psychiatrists in the clinical diagnosis and treatment of MDD.

## Introduction

Major depressive disorder (MDD) is a general chronic psychiatric disorder affecting people of all ages, which can ultimately lead to chronic disability, financial difficulties, and shortened life expectancy (Murray and Lopez, 1997; Zhdanava et al., 2021). In recent years, as increasing importance has been placed on the treatment of mental illness, the proportion of patients seeking treatment for MDD rose from 43.5% in 2007–2008 to 52.9% in 2015–2016 (Rhee et al., 2020). In China, MDD is the most common mood disorder, with a lifetime prevalence of 3.4% and a prevalence of 2.1% at 12 months (Huang et al., 2019). However, the misdiagnosis rate of MDD can reach as high as 78%, and misdiagnosis often leads to improper treatment (Fernández et al., 2010). The early identification of MDD is particularly important; therefore, it is of great significance to identify new and feasible biomarkers for the early diagnosis and treatment of MDD.

Obtaining peripheral blood biomarkers is a more convenient and practical method of diagnosis than brain imaging or biopsy. Recently, many studies have focused on the use of mRNA expression data from peripheral blood groups to investigate the differential characteristics between patients with MDD and healthy populations. For example, Woo et al. (2018) analyzed gene expression in peripheral blood of 38 patients with MDD and 14 healthy controls and identified seven differentially expressed genes (DEGs). Spijker et al. (2010) studied the peripheral blood of 21 patients with MDD and 21 healthy control participants and found significant differences in the expression levels of CAPRIN1, CLEC4A, CKRT23, MLC1, PLSCR1, PROK2, and ZBTB16, indicating that this signature could distinguish patients with depression from healthy individuals. Therefore, a predictive diagnostic model based on mRNA expression could help us understand the potential pathophysiology of MDD and may further support clinical decisions.

Currently, machine learning is increasingly applied in the medical field and has come to play an important role in the diagnosis and prognosis in the fields of oncology, neurology, and cardiology. For example, Ciobanu et al. (2020) used a random forest (RF) approach to predict depression and suicide risk in 39 patients with depression and 87 healthy controls using blood methylation and transcriptomic data, with an accuracy of 87.3% in distinguishing between the two groups. Bill et al. used a regularization gradient enhancement machine to classify microarray gene expression data in the blood of 1581 patients with MDD and 369 controls, with an average area under the curve (AUC) of 0.64 (Qi et al., 2021). An artificial neural network (ANN) model has further been applied in the diagnosis of many asymptomatic and early diseases (He et al., 2020) using a neural network classifier to the voice of patients with depression. The control group variables were analyzed, and the diagnostic accuracy rate was between 82.40 and 93.02% (Navarro et al., 2019). ANNs can accurately predict the positive or negative effects of Alzheimer’s disease and *Mycobacterium tuberculosis*, with a total accuracy of 93.8 and 94% (Khan et al., 2019; Swietlik and Bialowas, 2019). However, no ANN-related diagnostic model has been applied to construct an auxiliary peripheral blood diagnostic model for patients with MDD.

In this study, we obtained the MDD-related datasets, GSE98793 and GSE19738, and analyzed DEGs in patients with MDD. Functional analyses, including gene ontology (GO) and Kyoto Encyclopedia of Genes and Genomes (KEGG), were performed to investigate the enriched molecular functions and pathways. Machine learning algorithms, including RF and ANN, were used for variable selection and diagnostic model construction. Based on these results, we compared the discrimination and accuracy of single genes and diagnostic models for MDD.

## Materials and methods

### Data collection and data processing

The GSE98793 and GSE19738 datasets were searched from the Gene Expression Omnibus database^1^ using the following keywords: “MDD, blood, normal” [All Fields] AND “Homo sapiens” AND “Expression profiling by array” [All Fields]. The screening standards for microarray datasets included the following: reference to profiles of gene expression with genome-wide whole blood; containing samples from patients with MDD and healthy controls; all included samples were not treated with drugs; the number of samples was greater than 40. Eventually, GSE98793 (Leday G. G. R. et al., 2018) and GSE19738 (Spijker et al., 2010) were screened for in-depth investigation. The GSE98793 dataset was provided by Kelly et al., who examined a total of 192 peripheral blood samples, including 128 from MDD patients and 64 from healthy volunteers. And Affymetrix Human Genome U133 Plus 2.0 Array was used to test. GSE19738 data set was provided by Spijker, using the chip Agilent-012391 Whole Human Genome Oligo Microarray G4112A. They detected 132 peripheral blood samples in total from 34 healthy volunteers and 33 MDD patients, respectively. The information for patients with MDD and healthy participants was provided in Table 1.

**TABLE 1:** The information of datasets.

Dataset	Platform	Organism	Tissue	Sample	
				**Normal**	**Disease**
				
GSE19738	GPL6848	Homo Sapiens	Blood	34	33
GSE98793	GPL570	Homo Sapiens	Blood	64	128

### Differential expression analysis

The limma package in R software was used for standardized processing of the datasets to eliminate changes in gene expression caused by experimental techniques, and the normalized data were used for subsequent analysis. Differential analysis was carried out on the GSE19738 dataset, and the cutoff value was set to | log_2_FC| > 1, adj. *P* < 0.05.

### Functional enrichment analysis and protein-protein interaction network

To explore the function and pathways of the identified DEGs, GO and KEGG pathway enrichment analyses were performed using the clusterProfiler R package. Statistical significance was set at *P* < 0.05. The protein-protein interaction (PPI) network between the DEGs was analyzed using the STRING database^2^. The interaction score was set to 0.900 (highest confidence), and the nodes in the network were randomly clustered using the *k*-means algorithm to reveal potential regulatory relations between nodes. Subsequently, the ten most significant hub genes were screened using cytoHubba.

### Variable selection and diagnostic model construction

The RF algorithm is a classifier that contains multiple decision trees. It uses the RF package for analysis and sorts genes according to their importance. Genes with high importance were extracted from the list of different genes for visualization.

After the MDD characteristic genes were screened using the RF algorithm, information redundancy was removed by collinearity analysis. Taking the threshold of the Spearman Rho absolute value as >0.5, the parameters with collinearity were removed, and the model was further constructed using an ANN.

The ANN consists of the following three layers: the input, hidden, and output layers of six, five, and two neurons, respectively. An ANN software simulator was used to solve the return of the mission, including the forecast revision numbers. The network’s answer to each test case ranged from 0 to 1. The level of activation and inhibition of the output neurons is automatically selected by the stimulator of the ANN to minimize losses. The error function of the ANN was chosen as the sum of the square of the prior given value and the actual value of the output neuron. GSE19738 and GSE98793 were used as the training and test groups, respectively. GSE19738 was used in the initial receiver operating characteristic (ROC) curve analysis, ANN model predictive value of the MDD model. The GSE98793 dataset was used to test the model. The code is provided in Supplementary material 1 and Supplementary material 2.

### Immune cell infiltration analysis and correlation analysis

CIBERSORT^3^ and the LM22 characteristic gene matrix were used to predict the proportion of 22 immune cells in all samples of the dataset. The CIBERSORT package was used to assess the abundance of 22 immune cells in the GSE19738 dataset. Using the median prediction index of the ANN model as the cutoff value, the samples were divided into high-and low-score groups, and the differences in immune cell infiltration among the 22 groups were analyzed.

## Results

### Identification of differentially expressed genes and enrichment analyses

**FIGURE 1:**
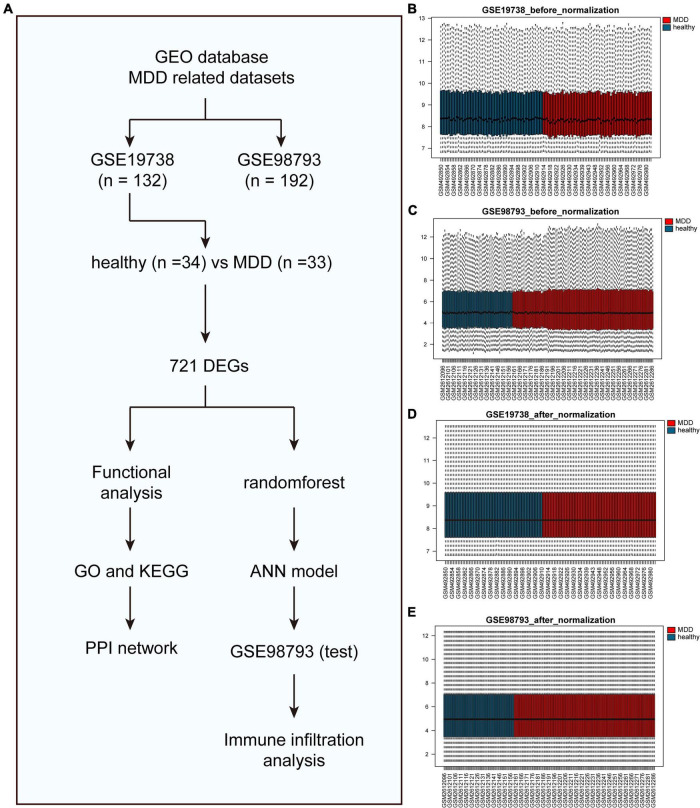
Before and after box diagram of standardization of GSE19738 and GSE98793 datasets. **(A)** The flow chart of this study. **(B)** Box diagram of the GSE19738 dataset before correction; **(C)** Box diagram of the GSE98793 dataset before correction; **(D)** Box diagram of the GSE19738 dataset after correction; and **(E)** Box diagram of the GSE98793 dataset after correction; Red represents the MDD samples, and blue represents the normal samples.

**FIGURE 2:**
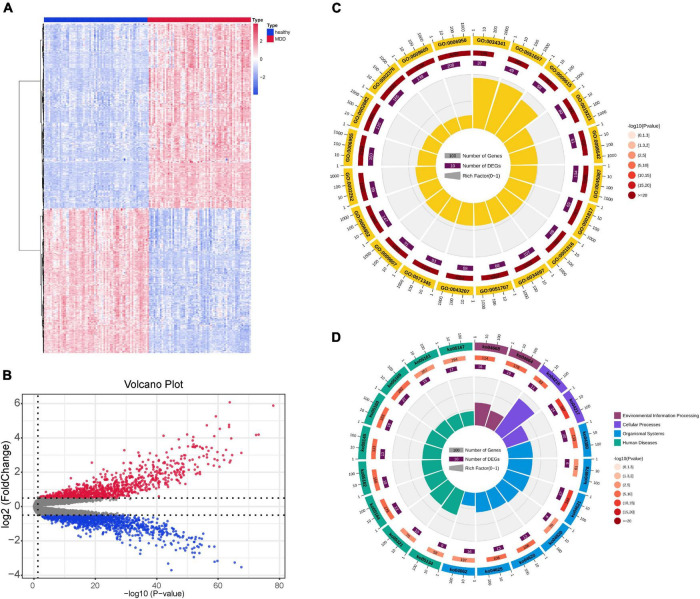
DEG identification of the GSE19738 dataset. **(A)** DEGs of peripheral blood samples from patients with MDD and healthy controls were obtained from the DEG heat map constructed from the GSE19738 dataset. Horizontal coordinate blue represents the control group, red represents the experimental group, blue indicates low expression, and red indicates high expression. **(B)** Volcano diagram, black indicates genes with no differential expression, blue indicates down-regulated genes, and red indicates up-regulated genes. **(C)** GO enrichment analysis. The outer circle represents the number of GO term, the outer circle number represents all genes in GO term, and the inner circle number represents the number of enriched genes. The inner circle pie chart represents the percentage of genes that are enriched. **(D)** KEGG pathways. The outer circle represents the KEGG ID, the outer circle number represents all genes in the KEGG pathway, and the inner circle number represents the number of genes enriched in the pathway. The inner circle pie chart represents the percentage of genes that are enriched. DEGs, differentially expressed genes; GO, Gene Ontology; KEGG, Kyoto Encyclopedia of Genes and Genomes.

A flowchart of this study is shown in Figure 1A. Figures 1B–E shows data distribution before and after standardization of data sets GSE19738 and GSE98793. First, compared to healthy participants, 721 genes were differentially expressed in the peripheral blood samples of patients with MDD. Among them, 404 DEGs were upregulated, and 317 DEGs were downregulated. The corresponding volcano and heat maps are shown in Figures 2A,B.

By analyzing the GO and KEGG pathway enrichment analyses, MDD peripheral blood raises the biological function of genes. The results of GO annotation revealed that the DEGs mainly comprised genes related to cytokines, defense responses to viruses, responses to biotic stimuli, immune effector processes, and responses to external biotic stimuli (Figure 2C). Prior studies have suggested that abnormal cytokine homeostasis may be related to the pathogenesis of MDD and that cytokine profiles may be used to distinguish patients with MDD (Petralia et al., 2020). KEGG signal pathway enrichment analysis results showed that the DEGs were mainly enriched in the immune system, cell growth and death, development, and infectious diseases (Figure 2D). MDD is associated with proinflammatory activation of the peripheral innate immune system, coupled with relative inactivation of the adaptive immune system (Leday G. et al., 2018). Several growth factors have been shown to play important roles in cell survival, growth, programmed death, and neuroplasticity and are associated with MDD (Li et al., 2021). The detailed data are presented in Tables 2, 3.

**TABLE 2:** GO enrichment analyses results.

GO ID	Description	Number	*P* value	*Q* value
GO: 0034341	Response to interferon-gamma	37	1.46E-24	4.15E-22
GO: 0051607	Defense response to virus	49	3.74E-32	2.36E-29
GO:0009615	Response to virus	58	5.70E-35	4.63E-32
GO:0019221	Cytokine-mediated signaling pathway	80	2.27E-32	1.61E-29
GO:0098542	Defense response to other organism	61	1.42E-24	4.15E-22
GO:0045087	Innate immune response	104	8.81E-42	1.25E-38
GO:0001817	Regulation of cytokine production	69	2.04E-25	6.84E-23
GO:0001816	Cytokine production	69	3.01E-25	9.51E-23
GO:0034097	Response to cytokine	107	2.77E-39	2.62E-36
GO:0051707	Response to other organism	88	7.14E-30	3.11E-27
GO:0043207	Response to external biotic stimulus	88	7.66E-30	3.11E-27
GO:0071345	Cellular response to cytokine stimulus	93	1.43E-31	8.12E-29
GO:0009607	Response to biotic stimulus	91	8.60E-31	4.45E-28
GO:0006952	Defense response	147	2.56E-47	1.46E-43
GO:0002252	Immune effector process	99	4.33E-30	2.05E-27
GO:0006955	Immune response	160	1.07E-45	3.03E-42
GO:0002682	Regulation of immune system process	110	8.62E-26	3.06E-23
GO:0002376	Immune system process	185	1.02E-42	1.93E-39
GO:0009605	Response to external stimulus	139	7.34E-26	2.78E-23
GO:0006950	Response to stress	208	1.86E-39	2.12E-36

**TABLE 3:** KEGG enrichment analyses results.

KEGG ID	Description	Number	*P* value	*Q* value
ko04668	TNF signaling pathway	16	4.09E-08	1.39E-06
ko04064	NF-kappa B signaling pathway	19	1.94E-07	5.11E-06
ko04216	Ferroptosis	11	9.06E-09	3.58E-07
ko04217	Necroptosis	24	8.24E-12	9.77E-10
ko04621	NOD-like receptor signaling pathway	27	2.20E-13	5.22E-11
ko04380	Osteoclast differentiation	20	2.17E-10	1.71E-08
ko05164	Influenza A	22	1.40E-09	8.30E-08
ko05169	Epstein-Barr virus infection	27	4.83E-09	2.29E-07
ko05162	Measles	18	6.86E-08	2.03E-06
ko05160	Hepatitis C	18	2.47E-07	5.85E-06
ko04620	Toll-like receptor signaling pathway	13	4.66E-06	9.21E-05
ko04625	C-type lectin receptor signaling pathway	13	4.66E-06	9.21E-05
ko05145	Toxoplasmosis	13	7.74E-06	1.41E-04
ko05321	Inflammatiory bowel disease (IBD)	10	1.31E-05	2.07E-04
ko05167	Kaposi sarcoma-associated herpesvirus infection	17	1.31E-05	2.07E-04
ko05134	Legionellosis	9	1.80E-05	2.67E-04
ko04978	Mineral absorption	9	2.74E-05	3.82E-04
ko05161	Hepatitis B	15	3.21E-05	4.23E-04
ko04062	Chemokine signaling pathway	16	6.01E-05	7.50E-04
ko04920	Adipocytokine signaling pathway	9	8.36E-05	9.90E-04

### Construction of protein-protein interaction network

**FIGURE 3:**
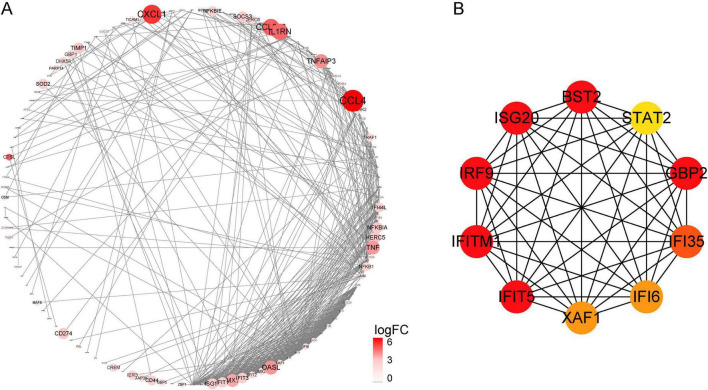
**(A)** Protein-protein interaction network of DEGs constructed using cytoscape. **(B)** The top 10 hub genes were explored using CytoHubba.

To further understand the relationship between DEGs at the protein level, we built a PPI network using the STRING database, which contains 487 nodes and 886 edges (Figure 3A). CytoHubba plugins were used to screen the top 10 hub genes, namely *BST2, STAT2, GBP2, IFI35, IFI6, XAF1, IFIT5, IFITM1, IRF9*, and *ISG20* (Figure 3B). Among them, dysregulation of the GBP2 gene could indicate a relationship between cell surface receptors and intracellular effectors that can transmit extracellular information into cells, as well as an intracellular signal transduction protein (Jiang et al., 2015). Furthermore, the proteins encoded by IFI6 may play an important role in the regulation of apoptosis and restrict various viral infections by targeting different stages of the viral life cycle (Sajid et al., 2021).

### Random forest screening of characteristic genes and major depressive disorder diagnostic model construction and validation

**FIGURE 4:**
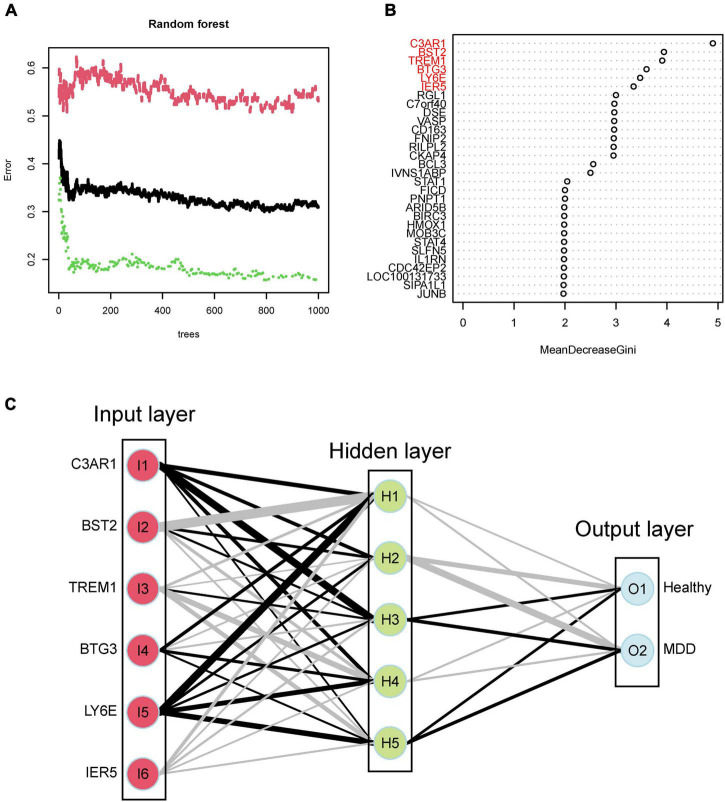
**(A)** Random forest tree. **(B)** MeanDecreaseGini. **(C)** Artificial Neural Network model. Healthy stands for healthy group, and MDD stands for MDD group.

**FIGURE 5:**
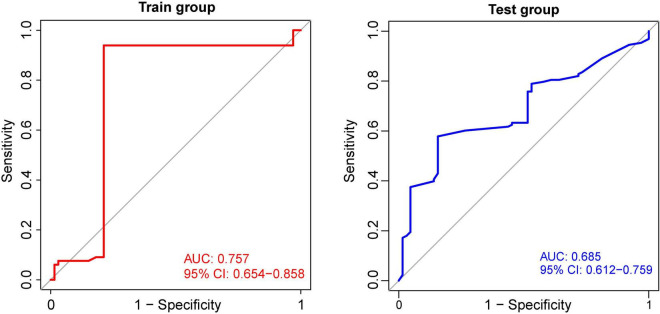
Receiver operating characteristic curves for the artificial neural network. The AUC curve of GSE19738 training cohort is on the left, and the AUC curve of GSE98793 testing cohort is on the right.

Using the identified DEGs, we further screened MDD-related characteristic genes using the RF algorithm. Six characteristic genes related to MDD were screened according to a gene score of >3 (Figures 4A,B). Also, we tested the correlation between these genes, and found there was no significant covariance between them (Supplementary Table 1). Furthermore, based on the features of the six MDD-related genes, we built an ANN model. The MDD diagnostic model constructed using the ANN includes the input, hidden, and output layers, as shown in Figure 4C. Among them, the dimension of the input vector is six, and the dimensions of the output vector of the control and disease. Based on the scoring values of the ANN model, ROC analysis was performed on the model to verify its accuracy. As shown in Figure 5, the AUC was 0.757 in the training set, indicating good accuracy. In addition, the accuracy of the model was further tested using the GSE98793 dataset, and the AUC of the test set was 0.685, indicating the high accuracy of the model.

### Immune infiltration and correlation analysis

**FIGURE 6:**
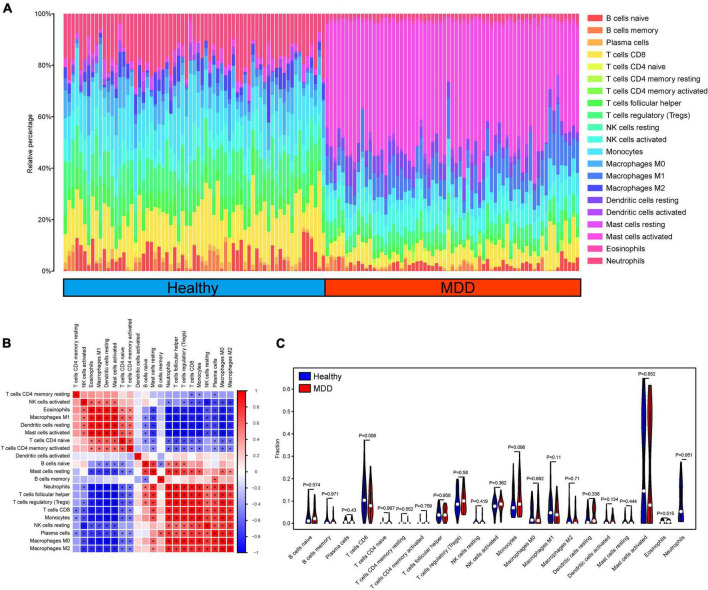
Evaluation and correlation analysis of immune cell infiltration. **(A)** Panoramic view of 22 immune cell infiltrates in peripheral blood samples; **(B, C)** High and low expression group of immune cell infiltration difference.

To analyze the relationship between the ANN model and immune cell infiltration, the CIBERSORT algorithm was used to calculate the proportion of immune cell infiltration in the peripheral blood of the healthy and MDD groups. In the immune analysis, we drew the immune landscape of infiltrated immune cells of patients with MDD and normal volunteers (Figure 6A). We found that a variety of immune cells were significantly correlated in the disease group (Figures 6B,C). However, we did not find any significant differences in the immune cells between the MDD and normal groups. This may be related to the sources of the specimens used in this study.

## Discussion

Patients with depression have a high rate of misdiagnosis; as such, it is important to find new and feasible biomarkers to facilitate the early diagnosis and treatment of MDD. Previous studies have reported a variety of algorithms used to construct diagnostic models for patients with MDD. However, at present, there are no reports on the application of ANNs to construct auxiliary peripheral blood diagnostic models for patients with MDD. Here, we tried to find diagnostic markers related to MDD and applied ANN machine learning methods to construct diagnostic models to explore better diagnostic models for patients with MDD that are suitable for different populations.

We identified 404 DEGs in the peripheral blood of patients with MDD compared to healthy controls. Through enrichment analysis of GO and KEGG pathways, we further identified that the biological functions of upregulated genes in the peripheral blood of patients with MDD mainly targeted cytokines, defense response to viruses, response to biotic stimulus, immune effector process, response to external biotic stimulus, response to external stimulus and immune system, cell growth and death, development, infectious diseases, and other pathways. We constructed a PPI network to screen out the top 10 hub genes, namely *BST2, STAT2, GBP2, IFI35, IFI6, XAF1, IFIT5, IFITM1, IRF9*, and *ISG20*. Moreover, we selected the following six characteristic genes in order of importance by RF algorithm: *C3AR1, BST2, TREM1, BTG3, LY6E*, and *IER5*. C3AR1 is a critical regulator of central immune homeostasis in tau pathology whose signaling operates intracellularly in human CD4 + cells and participates in several T-cell functions (Arbore et al., 2016). Litvinchuk et al. (2018) showed that the expression of the C3a receptor (*C3AR1*) is positively correlated with cognitive decline and Braak staging in human Alzheimer’s disease brains. BST2 has been identified as a marker of immunomodulatory bone marrow mesenchymal stem cell cloning, as well as an effective inhibitor of enveloped virus release (James et al., 2015). TREM-1 is an activating receptor expressed at high levels in neutrophils and monocytes that infiltrate human tissues infected with bacteria. Furthermore, it is upregulated in peritoneal neutrophils of patients with microbial sepsis and mice with experimental lipopolysaccharide-induced shock (Bouchon et al., 2001). Chronic stress contributes to the development of psychiatric disorders, including anxiety and depression. Mouse hippocampal RNA sequences showed that stress increases the TREM1 pathway associated with inflammation (DiSabato et al., 2021). Monocytes are in a pro-inflammatory state in patients with severe psychiatric disease. The expression of TREM-1 is significantly increased in monocytes of patients with SCZ and BD and tends to be overexpressed in patients with major depression (Weigelt et al., 2011). Regulating the imbalance in TREM1 expression ameliorates depression-like behaviors and impairment of learning and memory in rats (Fang et al., 2019). In addition, Rosie Owens suggested that neuroinflammatory conditions that alter the balance of TREM1 expression may be important factors affecting microglial inflammation and homeostasis activity and may be associated with neuroinflammation and neurodegenerative disease (Owens et al., 2017). BTG3 is a member of the anti-proliferative protein family. IER5 may play an important role in mediating the cellular response to mitogenic signals (Williams et al., 1999). Savitz et al. found using genome-wide expression analysis of peripheral blood mononuclear cells that IER5 was differentially expressed between 29 unmedicated depressed patients with a mood disorder (8 bipolar disorder and 21 MDD) compared to 24 healthy controls (Savitz et al., 2013). These differentially upregulated genes were closely related to MDD and mental and neurological diseases.

Based on the application of the above six characteristic genes using the ANN method, we successfully established an ANN model and further calculated the infiltration of two groups of immune cells in peripheral blood. In the training set, the AUC was 0.757, indicating good accuracy. The accuracy of the model was further tested, and the AUC of the test set was calculated to be 0.685. The reason of that the AUC is less than 0.7 may be due to the small sample size in the test set. At present, many studies have reported the application of RF, support vector machine, *k*-nearest neighbors (kNN), and naive bayesian (NB) algorithms to build diagnostic models for patients with MDD. These studies have reported high classification accuracies ranging from 70 to 100% (Yi et al., 2012; Yu et al., 2016; Bhak et al., 2019). In particular, Zhao et al. compared different machine learning approaches using the same data set which also used by us. It was found that compared with other methods, such as SVM, RF, kNN, NB, SVM could distinguish MDD from healthy controls more accurately (Zhao et al., 2021). Overall, compared with previous studies, our model evaluation provides new ideas for the application of peripheral blood in aiding diagnostic machine learning. ANNs are the most common form of neuromorphic computing, and breakthrough progress has been made in many areas. Neural networks are composed of multiple layers, each made up of a collection of cells called artificial neurons that are connected by artificial synapses (Subbulakshmi Radhakrishnan et al., 2021). One difference in our study is that upregulated differential genes are used in the analysis of differences between patients with MDD and control groups as clinical indicators tend to focus more on increased indicators, and upregulated genes can be more effectively applied to the analysis of blood indicators in outpatient and inpatient patients. In previously reported studies, although cross-validation from the same dataset can also be used for model validation, compared with the external validation with completely independent data, the previous experiments do not reflect the universality or replicability of the model (Steyerberg and Harrell, 2016). In this study, we used previously unused datasets to calculate the AUC of the classifier.

In this study, an ANN was used for the first time to establish an auxiliary diagnosis model of MDD, which provides a new method for MDD diagnosis using machine learning and could help clinicians reduce the misdiagnosis rate of MDD. However, this study had several limitations. First, the data used in this study were obtained from public databases. They did not provide comprehensive clinical information, such as age, sex, and BMI, which should be controlled. Meanwhile, transcriptome data are the main data in GEO database in the field of mental disorder, other types of data such as genomic and proteomic data, are lacking. Second, compared with traditional methods, such as logistic regression, the ANN method cannot obtain an image of the patient score for each variable. Third, the sample size of this study was relatively small; the more samples the machine learning model is subjected to, the more similar the sample sources are, and the more accurate the model is constructed. Fourth, due to the relatively small sample size of mental diseases in the public database, the potential transcriptome biomarkers for mental disorders was not well studied, so further comparison was not conducted between MDD and other mental disorders. In addition, the MDD samples we selected were not divided into subtypes and may have different clinical characteristics. As such, the representativeness and extensibility of the model will be limited to some extent. In future research, we will further explore the differential gene of MDD, expand the sample size, collect our own data, consider the influence of clinical features and subtypes of MDD. In order to find better diagnostic models for patients with MDD, we will compare more machine learning method such as SVM, RF, kNN, NB, etc. And continue to optimize and improve the model design to provide more reliable auxiliary tools for the diagnosis of MDD.

## Conclusion

In conclusion, we adopted a popular machine learning algorithm, RFs, and ANNs to filter the characteristics of patients with MDD and construct a diagnostic model. This model was then verified in an external test set. This validation established that this model could clearly distinguish between patients with MDD and healthy controls. This model could serve as a potential adjunct tool to help psychiatrists make clinical diagnoses and treatment plans.

## Data availability statement

Publicly available datasets were analyzed in this study. This data can be found from the Gene Expression Omnibus: https://www.ncbi.nlm.nih.gov/geo/, GSE19738 and GSE98793 datasets.

## Author contributions

SL and TL collected the data, conducted the analysis, and drafted the manuscript. QZ, BF, GL, FY, and JH conducted the data analysis and helped to interpret the results. HW and NL revised the manuscript. All authors contributed to the article and approved the submitted version.

## Footnotes

1
https://www.ncbi.nlm.nih.gov/geo/

2
https://string-db.Org

3
http://CIBERSORT.stanford.edu/

## Supplementary material

Supplementary Table 1 Correlation analysis between features genes.

Supplementary Material 1 The code of Artificial Neural Network (ANN) model.

Supplementary Material 2 The code of Random Forest (RF) model.

## References

[B1] Arbore G. West E. Spolski R. Robertson A. Klos A. Rheinheimer C. (2016). T helper 1 immunity requires complement-driven NLRP3 inflammasome activity in CD4+ T cells. *Science* 352:aad1210. 10.1126/science.aad1210 27313051 PMC5015487

[B2] Bhak Y. Jeong H. Cho Y. Jeon S. Cho J. Gim J. (2019). Depression and suicide risk prediction models using blood-derived multi-omics data. *Transl. Psychiatry* 9:262. 10.1038/s41398-019-0595-2 31624227 PMC6797735

[B3] Bouchon A. Facchetti F. Weigand M. Colonna M. (2001). TREM-1 amplifies inflammation and is a crucial mediator of septic shock. *Nature* 410 1103–1107. 10.1038/35074114 11323674

[B4] Ciobanu L. G. Sachdev P. S. Trollor J. N. Reppermund S. Thalamuthu A. Mather K. A. (2020). Downregulated transferrin receptor in the blood predicts recurrent MDD in the elderly cohort: a fuzzy forests approach. *J. Affect Disord.* 267 42–48. 10.1016/j.jad.2020.02.001 32063571

[B5] DiSabato D. Nemeth D. Liu X. Witcher K. O’Neil S. Oliver B. (2021). Interleukin-1 receptor on hippocampal neurons drives social withdrawal and cognitive deficits after chronic social stress. *Mol. Psychiatry* 26 4770–4782. 10.1038/s41380-020-0788-3 32444870 PMC8730339

[B6] Fang K. Li H. Chen X. Gao X. Huang L. Du A. (2019). viaQuercetin Alleviates LPS-Induced Depression-Like Behavior in Rats Regulating BDNF-Related Imbalance of Copine 6 and TREM1/2 in the Hippocampus and PFC. *Front. Pharmacol.* 10:1544. 10.3389/fphar.2019.01544 32009956 PMC6978986

[B7] Fernández A. Pinto-Meza A. Bellón J. Roura-Poch P. Haro J. Autonell J. (2010). Is major depression adequately diagnosed and treated by general practitioners? Results from an epidemiological study. *Gen. Hosp. Psychiatry* 32 201–209. 10.1016/j.genhosppsych.2009.11.015 20302995

[B8] He R. Zhang W. Chen S. Liu Y. Yang W. Li J. (2020). Transcriptional profiling reveals the regulatory role of DNER in promoting pancreatic neuroendocrine neoplasms. *Front. Genet.* 11:587402. 10.3389/fgene.2020.587402 33329729 PMC7728999

[B9] Huang Y. Wang Y. Wang H. Liu Z. Yu X. Yan J. (2019). Prevalence of mental disorders in China: a cross-sectional epidemiological study. *Lancet Psychiatry* 6 211–224. 10.1016/s2215-0366(18)30511-x 30792114

[B10] James S. Fox J. Afsari F. Lee J. Clough S. Knight C. (2015). Multiparameter analysis of human bone marrow stromal cells identifies distinct immunomodulatory and differentiation-competent subtypes. *Stem Cell Rep.* 4 1004–1015. 10.1016/j.stemcr.2015.05.005 26070611 PMC4471830

[B11] Jiang Y. Wu R. Chen C. You Z. Luo X. Wang X. (2015). Six novel rare non-synonymous mutations for migraine without aura identified by exome sequencing. *J. Neurogenet.* 29 188–194. 10.3109/01677063.2015.1122787 26814133

[B12] Khan M. T. Kaushik A. C. Ji L. Malik S. I. Ali S. Wei D. Q. (2019). Artificial neural networks for prediction of tuberculosis disease. *Front. Microbiol.* 10:395. 10.3389/fmicb.2019.00395 30886608 PMC6409348

[B13] Leday G. Vértes P. Richardson S. Greene J. Regan T. Khan S. (2018). Replicable and coupled changes in innate and adaptive immune gene expression in two case-control studies of blood microarrays in major depressive disorder. *Biolo. Psychiatry* 83 70–80. 10.1016/j.biopsych.2017.01.021 PMC5720346 28688579

[B14] Leday G. G. R. Vertes P. E. Richardson S. Greene J. R. Regan T. Khan S. (2018). Replicable and coupled changes in innate and adaptive immune gene expression in two case-control studies of blood microarrays in major depressive disorder. *Biol. Psychiatry* 83 70–80. 10.1016/j.biopsych.2017.01.021 28688579 PMC5720346

[B15] Li Y. Jia Y. Wang D. Zhuang X. Li Y. Guo C. (2021). Programmed cell death 4 as an endogenous suppressor of BDNF translation is involved in stress-induced depression. *Mol. Psychiatry* 26 2316–2333. 10.1038/s41380-020-0692-x 32203159 PMC8440200

[B16] Litvinchuk A. Wan Y. Swartzlander D. Chen F. Cole A. Propson N. (2018). Complement C3aR Inactivation attenuates tau pathology and reverses an immune network deregulated in tauopathy models and Alzheimer’s Disease. *Neuron* 100 1337–1353. 10.1016/j.neuron.2018.10.031 30415998 PMC6309202

[B17] Murray C. Lopez A. (1997). Alternative projections of mortality and disability by cause 1990-2020: Global Burden of Disease Study. *Lancet* 349 1498–1504. 10.1016/s0140-6736(96)07492-2 9167458

[B18] Navarro J. Fernandez Rosell M. Castellanos A. Del Moral R. Lahoz-Beltra R. Marijuan P. C. (2019). Plausibility of a neural network classifier-based neuroprosthesis for depression detection via laughter records. *Front. Neurosci.* 13:267. 10.3389/fnins.2019.00267 30949025 PMC6437104

[B19] Owens R. Grabert K. Davies C. Alfieri A. Antel J. Healy L. (2017). Divergent Neuroinflammatory Regulation of Microglial TREM Expression and Involvement of NF-κB. *Front. Cell. Neurosci.* 11:56. 10.3389/fncel.2017.00056 28303091 PMC5332401

[B20] Petralia M. Mazzon E. Fagone P. Basile M. Lenzo V. Quattropani M. (2020). The cytokine network in the pathogenesis of major depressive disorder. Close to translation? *Autoimmun. Rev.* 19:102504. 10.1016/j.autrev.2020.102504 32173514

[B21] Qi B. Ramamurthy J. Bennani I. Trakadis Y. J. (2021). Machine learning and bioinformatic analysis of brain and blood mRNA profiles in major depressive disorder: A case-control study. *Am. J. Med. Genet B. Neuropsychiatr. Genet.* 186 101–112. 10.1002/ajmg.b.32839 33645908

[B22] Rhee T. G. Wilkinson S. T. Steffens D. C. Rosenheck R. A. Olfson M. (2020). Prevalence of treatment for depression among us adults who screen positive for depression, 2007-2016. *JAMA Psychiatry* 77 1193–1195. 10.1001/jamapsychiatry.2020.1818 32609306 PMC7330829

[B23] Sajid M. Ullah H. Yan K. He M. Feng J. Shereen M. (2021). The functional and antiviral activity of interferon alpha-inducible ifi6 against hepatitis b virus replication and gene expression. *Front. Immunol.* 12:634937. 10.3389/fimmu.2021.634937 33868257 PMC8047077

[B24] Savitz J. Frank M. Victor T. Bebak M. Marino J. Bellgowan P. (2013). Inflammation and neurological disease-related genes are differentially expressed in depressed patients with mood disorders and correlate with morphometric and functional imaging abnormalities. *Brain Behav. Immun.* 31 161–171. 10.1016/j.bbi.2012.10.007 23064081 PMC3577998

[B25] Spijker S. Van Zanten J. S. De Jong S. Penninx B. W. van Dyck R. Zitman F. G. (2010). Stimulated gene expression profiles as a blood marker of major depressive disorder. *Biol. Psychiatry* 68 179–186. 10.1016/j.biopsych.2010.03.017 20471630

[B26] Steyerberg E. Harrell F. (2016). Prediction models need appropriate internal, internal-external, and external validation. *J. Clin. Epidemiol.* 69 245–247. 10.1016/j.jclinepi.2015.04.005 25981519 PMC5578404

[B27] Subbulakshmi Radhakrishnan S. Sebastian A. Oberoi A. Das S. Das S. (2021). A biomimetic neural encoder for spiking neural network. *Nat. Commun.* 12:2143. 10.1038/s41467-021-22332-8 33837210 PMC8035177

[B28] Swietlik D. Bialowas J. (2019). Application of artificial neural networks to identify Alzheimer’s Disease using cerebral perfusion SPECT data. *Int. J. Environ. Res. Public Health* 16:1303. 10.3390/ijerph16071303 30979022 PMC6479441

[B29] Weigelt K. Carvalho L. Drexhage R. Wijkhuijs A. de Wit H. van Beveren N. (2011). TREM-1 and DAP12 expression in monocytes of patients with severe psychiatric disorders. EGR3, ATF3 and PU.1 as important transcription factors. *Brain Behav. Immun.* 25 1162–1169. 10.1016/j.bbi.2011.03.006 21421043

[B30] Williams M. Lyu M. Yang Y. Lin E. Dunbrack R. Birren B. (1999). Ier5, a novel member of the slow-kinetics immediate-early genes. *Genomics* 55 327–334. 10.1006/geno.1998.5679 10049588

[B31] Woo H. Lim S. Myung W. Kim D. Lee S. (2018). Differentially expressed genes related to major depressive disorder and antidepressant response: genome-wide gene expression analysis. *Exp. Mol. Med.* 50 1–11. 10.1038/s12276-018-0123-0 30076325 PMC6076250

[B32] Yi Z. Li Z. Yu S. Yuan C. Hong W. Wang Z. (2012). Blood-based gene expression profiles models for classification of subsyndromal symptomatic depression and major depressive disorder. *PLoS One* 7:e31283. 10.1371/journal.pone.0031283 22348066 PMC3278427

[B33] Yu J. Xue A. Redei E. Bagheri N. (2016). A support vector machine model provides an accurate transcript-level-based diagnostic for major depressive disorder. *Transl. Psychiatry* 6:e931. 10.1038/tp.2016.198 27779627 PMC5290347

[B34] Zhao S. Bao Z. Zhao X. Xu M. Li M. D. Yang Z. (2021). Identification of diagnostic markers for major depressive disorder using machine learning methods. *Front. Neurosci.* 15:645998. 10.3389/fnins.2021.645998 34220416 PMC8249859

[B35] Zhdanava M. Pilon D. Ghelerter I. Chow W. Joshi K. Lefebvre P. (2021). The prevalence and national burden of treatment-resistant depression and major depressive disorder in the United States. *J. Clin. Psychiatry* 82:20m13699. 10.4088/JCP.20m13699 33989464

