# Supplementary material for: A machine learning model for predicting patients with major depressive disorder: A study based on transcriptomic data
Source: Front Neurosci. 2022 Aug 8;16:949609. doi: 10.3389/fnins.2022.949609 (PMC9393475; doi:10.3389/fnins.2022.949609)
Supplement: Supplementary Table 1 — Correlation analysis between features genes. [file Table_1.docx]

**Table S1. Correlation analysis between feature genes.**

|  | C3AR1 | BST2 | TREM1 | BTG3 | LY6E | IER5 |
| --- | --- | --- | --- | --- | --- | --- |
| C3AR1 | 0 | 0.217722823 | 0.216848881 | 0.241941903 | 0.210450334 | 0.252501269 |
| BST2 | 0.217722823 | 0 | 0.219700522 | 0.188495465 | 0.235032164 | 0.20615748 |
| TREM1 | 0.216848881 | 0.219700522 | 0 | 0.208245276 | 0.165196865 | 0.225360523 |
| BTG3 | 0.241941903 | 0.188495465 | 0.208245276 | 0 | 0.19441694 | 0.269431434 |
| LY6E | 0.210450334 | 0.235032164 | 0.165196865 | 0.19441694 | 0 | 0.189722548 |
| IER5 | 0.252501269 | 0.20615748 | 0.225360523 | 0.269431434 | 0.189722548 | 0 |
