# Supplementary material for: A machine learning model for predicting patients with major depressive disorder: A study based on transcriptomic data
Source: Front Neurosci. 2022 Aug 8;16:949609. doi: 10.3389/fnins.2022.949609 (PMC9393475; doi:10.3389/fnins.2022.949609)
Supplement: Supplementary Material 1 — The code of Artificial Neural Network (ANN) model. [file Data_Sheet_1.docx]

The code of Artificial Neural Network (ANN) model

#install.packages("neuralnet")

#install.packages("NeuralNetTools")

library(neuralnet)

library(NeuralNetTools)

set.seed(12345678)

inputFile="geneScore.txt"

data=read.table(inputFile, header=T, sep="\t", check.names=F, row.names=1)

data=as.data.frame(t(data))

group=gsub("(.*)\\_(.*)", "\\2", row.names(data))

data$con=ifelse(group=="con", 1, 0)

data$treat=ifelse(group=="treat", 1, 0)

fit=neuralnet(con+treat~., data, hidden=5)

fit$result.matrix

fit$weight

#plot(fit)

pdf(file="neuralnet.pdf", width=9, height=7)

plotnet(fit)

dev.off()

net.predict=compute(fit, data)$net.result

net.prediction=c("con", "treat")[apply(net.predict, 1, which.max)]

predict.table=table(group, net.prediction)

predict.table

conAccuracy=predict.table[1,1]/(predict.table[1,1]+predict.table[1,2])

treatAccuracy=predict.table[2,2]/(predict.table[2,1]+predict.table[2,2])

paste0("Con accuracy: ", sprintf("%.3f", conAccuracy))

paste0("Treat accuracy: ", sprintf("%.3f", treatAccuracy))

colnames(net.predict)=c("con", "treat")

outTab=rbind(id=colnames(net.predict), net.predict)

write.table(outTab, file="neural.predict.txt", sep="\t", quote=F, col.names=F)
