# Supplementary material for: A machine learning model for predicting patients with major depressive disorder: A study based on transcriptomic data
Source: Front Neurosci. 2022 Aug 8;16:949609. doi: 10.3389/fnins.2022.949609 (PMC9393475; doi:10.3389/fnins.2022.949609)
Supplement: Supplementary Material 2 — The code of Random Forest (RF) model. [file Data_Sheet_2.docx]

The code of Random Forest (RF) model

#install.packages("randomForest")

library(randomForest)

set.seed(123456)

library(tidyverse)

rt <- data.table::fread('GSE19738_norm.csv')

up_gene <- data.table::fread('up_dif.csv')

rt <- rt[rt$id %in% up_gene$symbol,]

rt <- rt %>% column_to_rownames(var ='id')

data=t(rt)

group=gsub("(.*)\\_(.*)", "\\2", row.names(data))

colnames(data)

rf=randomForest(as.factor(group)~., data=data, ntree=1000)

pdf(file="forest.pdf", width=6, height=6)

plot(rf, main="Random forest", lwd=2)

dev.off()

optionTrees=which.min(rf$err.rate[,1])

optionTrees

rf2=randomForest(as.factor(group)~., data=data, ntree=optionTrees)

importance=importance(x=rf2)

pdf(file="geneImportance.pdf", width=6.2, height=5.8)

varImpPlot(rf2, main="")

dev.off()

rfGenes=importance[order(importance[,"MeanDecreaseGini"], decreasing = TRUE),]

#rfGenes=names(rfGenes[rfGenes>2])

rfGenes=names(rfGenes[1:6])

write.table(rfGenes, file="rfGenes.txt", sep="\t", quote=F, col.names=F, row.names=F)

sigExp=t(data[,rfGenes])

sigExpOut=rbind(ID=colnames(sigExp),sigExp)

write.table(sigExpOut, file="rfGeneExp.txt", sep="\t", quote=F, col.names=F)

#if (!requireNamespace("BiocManager", quietly = TRUE))

# install.packages("BiocManager")

#BiocManager::install("limma")
